# Living with Diabetes: *Experiences of Inner and Outer Sources of Beliefs in Women with Low Socioeconomic Status*

**DOI:** 10.5539/gjhs.v8n8p200

**Published:** 2015-12-17

**Authors:** Wimonrut Boonsatean, Anna Carlsson, Margareta Östman, Irena Dychawy Rosner

**Affiliations:** 1 Faculty of Health and Society, Malmö University, Malmö, Sweden; 2 Faculty of Nursing Science, Rangsit University, Pathum Thani, Thailand

**Keywords:** Buddhist belief, low socioeconomic status, type 2 diabetes, vulnerability, woman’s health

## Abstract

The purpose of this study was to examine the life experiences of nineteen Thai women of low socioeconomic status who were living with type 2 diabetes. A qualitative research design was conducted, and the women were identified by the snowball technique. Data was collected through semi-structured interviews, and processes of induction and abstraction were used for data analysis. The theme “keeping equilibrium of one’s mind” involved two sub-themes: experiencing an unpredictable future and being empowered by emerged beliefs. The first sub-theme encompassed worries concerning health and fears of being a burden to one’s family. The second sub-theme comprised the experiences of continuing life without being conquered by the disease and believing in the natural law described in Buddhist teachings. These findings revealed that participants could maintain a balance among their concerns through empowerment by inner and outer sources of beliefs, particularly in Buddhist teachings. Despite the vulnerable situations caused by diabetes and low socioeconomic status, the women remained calm, with a consciousness to continue their lives with the disease. The Buddhist views on life, specifically natural law, assisted them to consider life with diabetes as simply a natural course. Buddhism served as a spiritual refuge and helped the women to cope with their psychological burden from diabetes. These findings may reflect the need for health care professionals to provide more holistic care that would assist patients to live with their disease. Buddhist beliefs can be used as a tool to assist Thai patients to empower themselves successfully.

## 1. Introduction

Type 2 diabetes (T2D) is influencing a growing number of people worldwide, inducing various complications that affect numerous aspects of a person’s life (Scobie & Samaras, 2012). The estimated global prevalence among adults aged 20-79 years for the year 2010 is 6.4% (Shaw, Sicree, & Zimmet, 2010). In Thailand, the scope of the problem is similar to these global trends, with approximately 7.7% of people being diagnosed (Shaw et al., 2010). In fact, a matter of rising concern for health professionals is to recognize and acknowledge how women who live with T2D perceive their illness because research has identified important differences between the genders in T2D development, with respect to high risk factors in women and their attitudes and beliefs regarding the disease (Tenzer-Iglesias, 2014). Recent studies report that women have poorer glycaemic control and physical function as a consequence of diabetes (Tenzer-Iglesias, 2014), as well as greater distress or depression, (Koch, Kralik, & Sonnack, 1999; Manderson & Kokanovic, 2009) than men have. Other studies show that women with diabetes have multiple responsibilities towards family members in addition to themselves (de Silva, Hegadoren, & Lasiuk, 2012; Penckofer, Ferrans, Velsor-Friedrich, & Savoy, 2007). The consequences of diabetes coexisting with the multiple roles and responsibilities of these women may affect their perception of life with diabetes and may influence their way of living.

Some investigators have explored issues related to how people with diabetes experience their lives when suffering from the disease (George & Thomas, 2010; Yamakawa & Makimoto, 2008). Research has found that people with diabetes perceive their lives dissimilarly because of a focus on certain matters. Aspects such as the impact of complications (Vermeire et al., 2007), the influences of illness on daily living and quality of life (George & Thomas, 2010), and the effect of the treatment costs on the family economy (Penckofer et al., 2007; Popoola, 2005) are all taken into account. Western studies have described the negative feelings that people living with T2D may have, such as feeling different from others (Vermeire et al., 2007) and being ashamed to talk about their disease (Utz et al., 2006). Furthermore, research has found a negative influence of the disease on family and social life (de Silva et al., 2012; Manderson & Kokanovic, 2009). However, a Thai study has found that women define themselves as being “normal” when they can work and perform daily activities of their usual lives even if they have diabetes (Naemiratch & Manderson, 2008). Another study of Thai Buddhist and Muslim women with T2D mentions religion as a significant factor that promotes psychosocial well-being when living with the disease (Lundberg & Thrakul, 2013), and one of the Buddhist teachings indicates illness as a common element in the cycle of human life (Phromtha, 1999). Additionally, research has reported that Thai people with diabetes believe in the consequence of actions, called karma, either in a past or a present life (Lundberg & Thrakul, 2012, 2013), and diabetes is acknowledged as a karma illness (Sowattanangoon, Kotchabhakdi, & Petrie, 2009).

The oft-mentioned lack of empirical studies that consider the lives of Asian people with diabetes (Li, Drury, & Taylor, 2013; Lundberg & Thrakul, 2013; Naemiratch & Manderson, 2008; Sowattanangoon et al., 2009; Yamakawa & Makimoto, 2008) and the high prevalence and morbidity of T2D as one of the top five chronic diseases in Thailand (Bureau of Epidemiology, Ministry of Public Health, 2013) encourage the understanding of how people perceive their lives with this disease. Moreover, the higher diabetes prevalence among Thai women makes it important to focus a study on their perceptions (Bureau of Epidemiology, Ministry of Public Health, 2013). While some research has considered women’s lives with diabetes in general (Li et al., 2013; Lundberg & Thrakul, 2013), almost none has focused on the perceptions of women with diabetes of low socioeconomic status. A narrow perspective on the various individual experiences may add to better understandings of how people in this population perceive their life situations with diabetes.

As a part of a wider study, the aim of this enquiry is to explore the life experiences of Thai women of low socioeconomic status who are suffering from T2D, specifically, experiences identified as meaningful and significant in their living with diabetes.

## 2. Method

A qualitative research approach inspired by Naturalistic inquiry was conducted in this study (Lincoln, 2007; Lincoln & Guba, 1985). This research approach pays attention not only to the population studied but also to the context of their living, their experiences and the meaning ascribed to them (Lincoln, 2007). This study used a descriptive style to describe Thai women’s own perceptions of their living with T2D and the ways to overcome the results of stressors.

### 2.1 Participants

Nineteen Thai Buddhist women with T2D who were between 40 and 73 years of age (mean age 59.5 years) were interviewed. Most participants had terminated their education at the primary school level. Nearly all participants were entitled to free health care under the universal coverage scheme or other social welfare programmes such as social security, except for one woman whose children paid for her treatment costs. All participants took one or more oral anti-diabetic agents. Each person had been diagnosed with T2D between 1 and 25 years previously (mean 9.2 years), and more than half of the participants had experienced hypoglycaemia or numbness of the hands and feet. Hypertension was their most common co-morbidity. The participants’ families usually combined the incomes earned by all family members and mostly spent these incomes on essential daily expenditures. Most of the women’s families earned just enough money to live on, namely, for spending on the basic daily costs of living for all family members; however, nearly one-third of the participants said they sometimes ran short on money and had to borrow money from relatives or neighbours. Nearly half of the participants did not work and received money and assistance from their children. Most women lived with their children in the same houses or had children or siblings living nearby in the same area. The general family positions of the participants were grandmother, mother, or wife; common roles were housekeepers or babysitters for grandchildren. All participants were Buddhists who believed in the fundamentals of Buddhism. Most of them prayed for a healthy and happy life for themselves and their families before going to bed. Sometimes, they participated in religious rites at a nearby temple.

### 2.2 Setting

The data for this study were collected in one community of the province, which was located close to Bangkok, Thailand. The area consisted of some people living in a substandard environment and others in slightly better circumstances. This setting was typified by three characteristics: 1) low income, 2) visibly poor accommodations, and 3) squatter residences. The land was owned by the Royal Irrigation Department, and consequently, the inhabitants lived there without having to pay rent. The possibility of eviction was unknown. All participants in our study were natives. Most of our interviewees had siblings or children (living in small houses nearby) who were their primary sources of assistance.

Many health care facilities (including private and public sectors) that provide various levels of health care are located around this research area. Thai people possess one type of preferential treatment, such as universal coverage, social security, or civil servant medical benefits, which specifies the name of the health facility used and referral hospitals-without payment for essential treatment costs. They can select their preferred hospitals if they can afford the cost of treatment. This entire setting is the responsibility of the Health Promoting Hospital (HPH), a frontline health care service engaged in proactive health promotion, preventive care, and primary medical care, as set forth by the Ministry of Public Health. Most inhabitants in this area were registered in the “universal coverage scheme”, which provides free essential health care (including diabetes care) at the HPH and a referral hospital. The health service for diabetes was offered as a special clinic once a week, managed by registered nurses under the supervision of a doctor from the Provincial hospital. This special clinic mostly focused on curative care, and diabetes education was arranged informally during a short contact between health care providers and participants.

### 2.3 Data Collection

Data was collected using the snowball technique and semi-structured interviews (Polit & Beck, 2010). The process began with two people, one over and one under the age of 60 years, who were considered representatives of the ages below and above retirement. Both interviewees were suggested by the management of the HPH. Each participant was asked to suggest another neighbour with T2D. If the qualification of that woman was found to be ineligible, another recommendation was requested. This process would be repeated until an eligible individual was found. There were three inclusion criteria for participation in this study: 1) Thai citizens, 2) a diagnosis of T2D made at least one year before the study, and 3) receiving one or more anti-diabetic agents, either orally or parenterally. All contacted women were eligible and participated throughout this study.

A trial of the interview questions was conducted with two people with T2D living in the same area. After a minor revision, the following open-ended question was developed: “Please tell me your experiences, your personal feelings and thoughts about the life with diabetes”. This question was followed by further questioning based on the responses, opening the way for the forthcoming dialogue.

One day before the interview, the first author visited the participant to explain the study, its purposes, advantages and disadvantages of the participation, and how to participate or withdraw from the study. The author also confirmed that participation was voluntary and set the interview date. At the interview, the research process was explained, permission to tape the conversation was requested, confidentiality of all data was assured, and an informed consent was signed. Each interview lasted between 50 and 90 minutes and was conducted in a friendly and non-threatening tone at the participant’s home or at a place chosen by the participant. Data from the audiotapes were later transcribed and translated into English, and the translations were rechecked by a bilingual language expert.

### 2.4 Data Analysis

The process of data analysis included the use of an inductive method to develop conclusions from the particular experiences related in the stories of participants to more general conceptualizations, followed by a process of construction going from manifest to abstract meaning, as suggested by Lincoln and Guba (1985). The first author read and re-read the transcripts to extract the dominant ideas from the narrative data and capture all meaningful units. Each co-author independently analyzed and identified categories to ensure trustworthiness (Shenton, 2004). Grouping homogeneous and differentiating heterogeneous content by meaning was performed and checked back to the transcribed text to confirm the meaning of the categories chosen. Finally, the categories were structured into themes and sub-themes with the collaboration of all authors through several discussions and revisions. Ultimately, the main findings were shown and discussed with the participants to ensure that the researchers correctly understood what the participants wanted to communicate.

### 2.5 Ethical Considerations

Approval to conduct this study was given by the medical officer committee of the Provincial Health Office, approval number 0027/3615. All participants were volunteers and were free to participate or withdraw at any time. Written informed consent was obtained in advance before the interview began. For three unschooled participants, thump-printing when illiterate is common practice in Thailand. The process started by the interviewer reading all the information in the consent form to them and provided the opportunity for questions. When the participants decided to participate in this study, they then stamped their thumb fingerprint on the form as an agreement to participate. Permission to audiotape the interviews was obtained prior to the commencement of each interview. All data collected and reported were treated confidentially.

## 3. Results

Women’s lives were revealed to be affected by T2D in many ways. The participants sensed that they were confronting susceptible conditions originated from an unpredictable future of having diabetes complications despite getting treatment. They feared living as dependent persons and being a burden to their families, which would make the participants feel useless. However, these feelings could be counterbalanced by their beliefs received from their strong minds and the gist of Buddhist teachings. These experiences were summarized in the theme of “keeping equilibrium of one’s mind.” It consisted of two sub-themes, experiencing an unpredictable future and being empowered by beliefs, and each sub-theme comprised two further categories.

### 3.1 Experiencing an Unpredictable Future

Diabetes gave most women the feeling that their future was unpredictable, even if they were receiving treatment and taking care of themselves. They felt uncertain about their health and unsure of their future situation. This left them with a feeling of vulnerability and a sense of dependency. Thus, the two categories that depicted their feelings when encountering this changeable future were worries concerning their health and fears of being a burden to their families.

#### 3.1.1 Worries Concerning Health

Living with diabetes led women to feel vulnerable when thinking of their ill-health in the future, and they also felt afraid to confront this prospect, as they knew that their disease was incurable and had a great potential for leading to unexpected or chronic conditions. Concerns about the state of their health and questions about longevity were predominant in their minds. Most participants were anxious about living with the common complications of diabetes in the future, especially blindness, leg amputations, or unconsciousness followed by paralysis. One woman expressed her concern about unpredictable conditions when living with diabetes: “I don’t feel stressed by having diabetes, but if I talk a lot about it, it makes me feel awful because I don’t know when it will make me faint. I may become unconscious at any time. I just don’t know.” Participants feared confronting the disease’s complications. They thought that their uncertain health would have a great impact on their personal and social lives, including daily living, social activities, or work. A woman expressed the wish for a sudden death, rather than facing the debilitating consequences of diabetes. “If I am unconscious and die, then I do not have to suffer. I want this to happen. However, if I become paralyzed, it makes a big difference. Oh! I cannot live in that situation. I often think of the day when I become unconscious because I am growing older and the disease is getting stronger.” This contemplation of death was brought about by woman’s susceptibility to the poor health she was experiencing and its potential effects on her everyday life.

#### 3.1.2 Fears of Being a Burden to Families

Fears of becoming a burden to family members were the feeling originated from a sense of dependency due to women’s inability, which was affected by diabetes complications. The feeling of being a burden drove the participants to feel useless because having such conditions would decrease their abilities to take care of themselves, causing them to rely on others to help them both physically and economically. The insecurity of becoming a dependent person was expressed by one woman who said, “Thinking about my unhealthy conditions makes me feel stressed about the future possibilities. If that happen, what will I do then? I will become a burden to my children and grandchildren and will need to be cared for by others.” Some participants perceived being a burden to their family members as a state of suffering. A woman with co-morbidities who had lost the ability to care for herself reflected on her negative experiences about living with diabetes, “The disease has taken my daughter’s money, and I cannot go anywhere by myself. My daughter has to clean my body and wipe my bottom. It’s such a troublesome task for her. I’m a burden to my daughter!” Her view also showed how helplessness led to a sense of dependency. By contrast, some participants attempted to maintain their health as well as they could with the goal of remaining independent for a long time. Due to their fearful feeling of becoming a burden, those women tried to keep themselves healthy by following the medical recommendations so that they could do everything they needed to do without causing any problems to their family members. One woman stated her intention not to allow herself to be a burden to her children: “I have to take very good care of myself, both for me and for my family … so my children don’t have to worry about me and won’t have any problems. If I’m sick and admitted to the hospital, at least one of my children has to stay and take care of me, according to the rules of the hospital. This makes it very tiring for them and interrupts their routines.” For several participants, the feelings of losing health and becoming a burden to their families often elicited anxiety and worries.

Some interviewees who lived in very needy circumstances had an extra worry about not contributing to their family income. If they had severe symptoms, they would then be dependent on their families in every respect—emotionally, practically, and economically. The participants’ primary concern was their household finances, rather than their health. As explained by a woman whose priority was her work, above the need of taking care of herself: “I rarely take care of myself. My work comes first. However, I worry about not being able to walk. Who will carry me to the bathroom? I cannot live in such a situation, so I never stop taking my medicines.” When participants had mild symptoms, they tolerated them until things became unbearable. They wanted to save the money they would need if they went to hospital. “I worry about myself. Don’t think that I am not worried! … If my condition gets so bad that I cannot tolerate it, I will tell my children. If I have mild symptoms and can bear them, I do not tell them because I do not want to burden them.” Another woman said, “I think if I die (from diabetes complication), that’s OK. That is better than dying of starvation.” This expression emphasized the importance of family finances, which were ranked first before the disease. Most women were afraid that one day they would become a burden to their families in various aspects, including physical, psychological, and financial. In that state, they perceived themselves as useless persons if they could not contribute to their family members’ well-being.

### 3.2 Being Empowered by Beliefs

Most women expressed a number of beliefs stemming from their own minds and from religious beliefs that kept their spirits up while living with T2D. Although they worried about the consequences of disease, they stayed calm and accepted their situation. Beliefs that empowered most women to possess a strong mind were described as two-fold: continuing life without being conquered by the disease and believing in the natural law as described in Buddhist teachings.

#### 3.2.1 Continuing Life without Being Conquered by Disease

The women continued their lives by telling themselves to have faith in the power of their minds to lead a good life despite having T2D. This approach helped them to be confident in their thoughts, decisions, and actions. The participants also motivated themselves not to give up and not to be discouraged or anxious because they believed that everything was dependent on their state of mind. A strong mind could strengthen their bodies. One participant reflected that a strong mind could overcome matters, even including the disease: “Some people believe in a doctor, but I believe in myself. Whether the diabetes will be severe or not depends on myself alone. If I have a strong mind, nothing can affect my body.” These participants believed that if their minds were powerful, they could make good decisions. A woman described her secret technique to build her mind to be forceful, “It’s most important to fight with your heart. Don’t be dispirited and don’t think that once you have diabetes you can never be cured or you will die. It depends on my mind. My life is in my own hands. It is up to me whether I survive or die.” Trust in the power of the mind was one thing that could lead the participants to continue their own lives in the way they chose and that would create their good lives. Thus, a strong mind could give them a sense of confidence and meaningfulness in living with diabetes.

#### 3.2.2 Believing in the Natural Law as Described in Buddhist Teachings

Women considered their lives with T2D as it was in the course of the natural truth. They believed that everything happened because it had one or more reasons to happen and that these occurrences would continue and change from day to day without being prevented by anyone. Holding their belief according to this rule helped the women remain composed and recognize what was transpiring in their minds and bodies. Such an attitude allowed them to visualize diabetes as an unavoidable and ordinary condition for them. Most participants believed that ageing, illness, and death were a natural part of human life that would proceed according to human destiny under the rules of nature. Some women mentioned their choice to accept T2D because they understood that their bodies had deteriorated due to ageing: “If diabetes occurs, it must be because I’m older.” “There is no choice. I must accept that it is because of my age.” Another woman stated a different explanation of why she could admit T2D into her life: “As humans, we must have a disease. If it is not one disease, it is another one. When I think of it this way, I can easily accept diabetes.” This way of thinking helped the women to accept their destiny.

The participants recognized human life as a continuous cycle through many lives, including the past life, the present life, and the next life. Women believed that having T2D in this life was the result of their deliberate actions from a previous life. They expressed acceptance of their condition because they perceived it as unchangeable. One woman stated her acceptance of the fact that she was destined to have T2D in this life whether its consequences were good or bad: “Diabetes will stick with me for my entire life. I think it is caused by my previous deeds. It does not get much better than this. It’s a human life cycle, so I don’t think about it… If I have it and become seriously ill, it is the retribution of mine from a former life, right?”

Accepting the rules of nature made the participants unafraid of death. They often talked about the possibility and recognized that one day they would reach that point. As a participant said, “Death is a part of being a human. Everyone has to die, whether slowly or quickly. That’s all.” Another woman exemplified this viewpoint by saying: “If it’s time to die, it will be. For human beings, when life is over, nothing can hold it back.”

Participants realized that their lives could not revert back to the time before they had T2D. “Get over it or let it go” was a general strategy used which women repeatedly told themselves about the natural truth of life with T2D according to the Buddhist teaching. It assisted women to accept the fact that T2D had already occurred and that they could not change this occurrence. They then should accept that T2D would remain with them for the rest of their lives. When the women could apply this method to their lives with the disease, it helped them to encounter and accept their situation calmly. After a certain period, some women could unconditionally admit the disease as being a part of themselves. It could be expressed by a participant thus: “Why should I suffer? I accept diabetes with all my heart. What will happen, will happen.” Such expressions were commonly used by most participants to encourage themselves to get over their situation, to neutralize their suffering, and to accept their condition compliantly. This attitude is exemplified by one participant’s comment: “I take many benefits from the earth, and this means I will live in this world for a long time. However, I cannot go against nature. If I am to have diabetes until I die, so be it, because I do have this disease. It doesn’t disappear. I don’t think about it. Let it be.” When the participants make sense and understood the meaning of their lives with T2D as something existing in the natural truth, this helped them accept any situations that were occurring or had already happened to them. They could remain calm whether confronting good or bad situations because they perceived that their conditions were ordinary and inevitable.

## 4. Discussion

The Thai women of low socioeconomic status living with T2D saw themselves as facing a partly unpredictable future against which they could be strengthened by their beliefs. The process of adaptation to live with T2D was revealed in their striving to balance their worries about health and being a burden to the family by the empowerment they received from their strong mind and Buddhist teachings that shaped their view of the universe. Buddhist beliefs gave them the power to lead a life of equanimity, despite their disease and its complications. 

The notion of unpredictable future complications and its consequences among people with T2D that we found in our study corresponds to contemporary research on other diabetes populations (Koch et al., 1999; Penckofer et al., 2007; Popoola, 2005). This notion may imply a general concern of people with diabetes that is related to the illness and may be a part of the medical knowledge they have received from health professionals. However, the worry about becoming a burden to family members is a noticeable finding in the current study. This feeling may be related to a cultural value in Thai society, namely, *krengjai*, meaning a consideration for other people’s feelings first and a refusal to worry other people with one’s own problems (Rabibhadana, 1999). Although Thai people are accustomed to family care and support in their daily lives, our study and earlier research showed that the *krengjai* feeling is also present in this relationship (Lundberg, 2000). According to Thai cultural value, to complain or ask for help from anyone (even family members) can cause trouble to other people and make the requester feel ill at ease. Although other people may be willing to help, the *krengjai* feeling still exists. Therefore, most Thai people would avoid requesting help from others, as found in our findings. The participants in this study tried instead to keep themselves healthy and independent so that they would not need to disturb their relatives’ routines.

Another reason for the fear of becoming a burden to the family may be the socioeconomic status of our participants. Due to their poverty level, the women attempt to live day to day within limited financial incomes. Thus, some women in this study and another investigation conducted in Thailand (Lundberg & Thrakul, 2012) focused on the survival of their family members rather than their health, as described in the literature regarding the culture of poverty (Larsen & Hardin, 2013). When our participants become dependent as a result of T2D, it would increase their experience of creating a family burden in many aspects. This may be one reason some women in this study attempted to keep themselves healthy as much as they could and tolerated mild symptoms as long as possible. These behaviours have the purpose of saving money that would have been spent on the cost of basic daily life that is not covered by the universal coverage scheme of the Thai health care system. At the same time, the behaviours adhered to the cultural value of *krengjai* that permeates Thai thought (Rabibhadana, 1999).

Our participants reflect equilibrium of mind by reducing their worries with their inner strengths and could maintain their present lives with the state of calmness. These findings contrast with studies conducted among Western women revealing distressed or depressed feelings due to living with diabetes (de Silva et al., 2012; Manderson & Kokanovic, 2009; Penckofer et al., 2007). Religious beliefs were shown to play an important role in assisting our participants and people with T2D in earlier studies to counterbalance their feelings when facing difficult situations (Gordon et al., 2002; Popoola, 2005). Buddhism is a way of life concerning the nature of being and knowledge, and the gist of Buddhist teaching helps people understand the nature of existence, their lives, and ways to find happiness (Srisopa, 2001). Holding this essence in Buddhism could assist our participants to view their lives with vulnerable conditions, including diabetes, socioeconomic status, and traditionally feminine roles in their families, as it is. They then acknowledge that everything that happens in their lives has one or more causes, which exist under the law of Karma. This philosophy explains that every action has a consequence, which may appear immediately after the action or wait until the next life to show its result; therefore, people reap what they sow (Phromtha, 1999; Podhisita, 1998). Hence, having diabetes in this life is inevitable, as some participants expressed that their disease was the retribution of their intentional bad deeds from past lives.

Realizing the natural truth enabled our participants to create a sense of mindfulness and accept their fates. This made the women aware of the present moment while calmly recognizing and accepting events that occur or will happen to them, and they finally chose to accommodate their disease as a part of their lives, with the aim to live with their disease and continue their usual lives. Hence, the participants could enable themselves to get over their situation, called *thahm jai* or *plong* in the Thai language. *Thahmjai* is an attempt to accept things that have already happened and *plong* allows individuals to successfully accept their disease from their hearts (Saihu, 1999). These approaches could assist the women to leave their psychological suffering behind and concentrate on the truth of the present situation. Then, they could encourage their heart to accept their situation calmly and pay no heed to the disease they have. These findings once more show the great influence of religious beliefs on strengthening our participants’ minds and demonstrate the effect of Buddhist teachings on the mental attitude of the women in our study.

Additionally, the women in this study perceived their lives with diabetes to be an ordinary condition for them. This viewpoint may be explained by the belief in *the round of existence*, meaning the cycle of human beings (comprising birth, ageing, illness, and death) that continues through many lives, without an ending point (Phromtha, 1999). Because illness as such is regarded as a natural part of human life, having diabetes appears acceptable for the participants. When women acknowledge these realities of life with diabetes, they can predict and prepare themselves for the future changes. The women perceive that they cannot go against the nature; thus, they select a way to stay calm with consciousness and to live with the disease. As for the women in this study, Buddhist beliefs appear to be a spiritual refuge that acts as a means of coping with T2D and a support in everyday life’s struggles, as consistently reported in earlier research (Lundberg & Thrakul, 2013; Sowattanangoon et al., 2009).

In accordance with other Western research (Hörnsten, Sandström, & Lundman, 2004), our participants believed that their diabetes was related to increasing age. However, the women in this study did not explain their belief according to medical knowledge as earlier found; instead they linked their belief to Buddhist teachings, namely, *the three common characteristics of existence*, which is included in the natural law. This notion states that suffering arises from the fact that everything in the world is impermanent and does not belongs to “me” forever (Podhisita, 1998). Believing in this notion, the participants understood that their bodies (diseased bodies) are liable to decay because of daily change and deterioration; thus, increasing age increases degeneration and induces more disease(s). Based on this belief in the natural truth, it is common for people to have diabetes. Death from diabetes is inescapable because people cannot own their bodies forever and one day will reach that point, as our participants expressed. In addition, our participants accepted the fact that death is a natural part of human life, as stated in *the round of existence*.

Interventions for patients with T2D in this population may require rethinking. It may be a challenge for health professionals such as nurses to be aware of the power of religious beliefs and the impact they can have on patients’ thoughts, feelings, and actions. There is a need to provide more holistic care, including bio-psychosocial aspects. Because the religious beliefs and cultural values facilitated the abilities of women in our study to cope with their lives with T2D, it is important for health professionals to incorporate a broader view of sociocultural facets in their treatment. A health intervention that utilizes Buddhist teachings to strengthen patients’ minds may empower them to cope with their situations more effectively.

### 4.1 Methodological Considerations

The study was conducted among women of low socioeconomic status in a suburban community near Bangkok, Thailand. Our findings may help elucidate the profound thinking and feelings of women with a low socioeconomic status in other suburban or rural zones of Thailand. This research may also benefit health professionals working with similar patients living in other low socioeconomic Buddhist areas.

Collecting data from people who had been diagnosed with T2D for at least one year may guarantee that the participants had adjusted themselves to their situations and presented the actual experiences of those affected by living with the disease. Moreover, the first author (WB), a Thai nurse researcher, conducted all the interviews, which may increase the credibility of the data (Shenton, 2004). Additionally, the interviewer understood the disease and was familiar with Thai culture. On the other hand, the professional status of nurses in Thai society might have induced the participants to be considerate of the nurse researcher’s feelings (*krengjai*), which might have resulted in the interviewees providing superficial information. To minimize this possibility, all interviewees were reassured that the first author was a researcher and that all data would be kept confidential and used only for this study. Furthermore, interviewing participants at their house or at the place and time they selected might have encouraged them to talk more freely about their experiences. The interviewer tried to reduce the risk of interruption during the interview by asking for permission to converse in a quiet place for at least one hour.

In the analysis phase, all authors independently read and analyzed the materials. They also participated in several revisions of the research themes, which may have increased the trustworthiness of the research (Shenton, 2004)

## 5. Conclusion

Our study focused on the experiences of Thai women living with T2D and with a low socioeconomic status. A major finding reflected the equilibrium between worries about an unpredictable future and empowerment though inner and outer sources of beliefs (one’s mind and Buddhist teaching, respectively) to accept diabetes as a part of their lives. The Thai cultural value, *krengjai*, and the socioeconomic status of the participants were important factors creating the fear of becoming a burden to the family. By contrast, Buddhist teachings played a significant role in creating a sense of mindfulness, encouraging the women to accept their destiny calmly and then put their worries or fears behind them in order to live with their disease despite their low socioeconomic status. These core findings may be useful for professionals in assisting patients to improve this balance by utilizing the power of Buddhist beliefs as an additional tool to help patients empower themselves.
